# Quantitative assessment of lesion load and efficacy of 3 cycles of albendazole in disseminated cysticercosis: a prospective evaluation

**DOI:** 10.1186/s12879-020-4891-5

**Published:** 2020-03-14

**Authors:** Sudhakar Pandey, Hardeep Singh Malhotra, Ravindra Kumar Garg, Kiran Preet Malhotra, Neeraj Kumar, Imran Rizvi, Amita Jain, Neera Kohli, Rajesh Verma, Praveen Sharma, Ravi Uniyal, Shweta Pandey

**Affiliations:** 1 Department of Neurology, King George’s Medical University, U.P, Lucknow, 226003 India; 2 Department of Pathology, R.M.L. Institute of Medical Sciences, Lucknow, 226010 India; 3 Department of Microbiology, King George’s Medical University, U.P, Lucknow, 226003 India; 4 Department of Radiodiagnosis, King George’s Medical University, U.P, Lucknow, 226003 India

**Keywords:** Disseminated cysticercosis, Neurocysticercosis, Albendazole, Corticosteroids, Lesion load

## Abstract

**Background:**

The management of disseminated cysticercosis is unclear and largely considered hazardous. The role of albendazole remains controversial in such patients.

**Methods:**

A tertiary care, University hospital-based prospective intervention study was conducted from December 2015 to December 2017. Patients with disseminated cysticercosis, defined as the presence of multiple viable neurocysticerci (≥ 3) in the brain along with involvement of an additional extra site, were included in the study. Patients with cysticercal encephalitis were excluded. A detailed evaluation, including ophthalmoscopy, ocular B scans, ultrasound abdomen, and X-rays were done. Albendazole was administered at a dose of 15 mg/kg/day in 3 cycles of 28 days each. All patients were also given adjuvant corticosteroids and anti-epileptic drugs. Clinical and radiological follow up was carried out at a difference of 3 months between each treatment cycle. For radiological quantification, lesions were counted at 10 pre-specified levels. Statistical analysis was done to estimate the difference in seizure frequency and lesion load.

**Results:**

Twenty-nine patients (21 with > 20 lesions; 8 with ≤ 20 lesions) were given albendazole as per the protocol. There was a significant reduction in the occurrence of seizures (*P* < 0.001) and headache (*P* < 0.001). A significant reduction in lesion load from baseline to third follow-up was seen in the estimations done at different levels (*P* < 0.001). No patient developed serious side-effect warranting cessation of therapy.

**Conclusion:**

Cyclical use of albendazole appears efficacious in treating disseminated cysticercosis. The method of quantification described may be used in future studies for objective assessment.

**Trial registration:**

ISRCTN11630542; 28th September 2019; Retrospectively registered.

## Background

Neurocysticercosis is amongst the most common causes of seizures, especially in developing countries like India. Neurocysticercosis is caused by the larval stage of *Taenia solium*. Clinically, cysticercal infections in humans can be divided into neurocysticercosis (infection of central nervous system by the larval stage of *T. solium*) and extra-neural cysticercosis (infection of other tissues like muscles, subcutaneous tissues, eyes) [1]**.** Disseminated cysticercosis is the systemic dissemination of cysticerci and includes neurocysticercosis along with extra-neural cysticercosis [2]**.** Disseminated cysticercosis is often referred to as an uncommon manifestation of a common disease and its treatment has been a bone of contention for long [3]**.** The clinical picture of cysticercosis depends upon the site, size, number of lesions as well as the inflammatory response mounted by the host [4]**.**

Randomized controlled trials have demonstrated that cysticidal agents like albendazole and praziquantel are safe and efficacious in treating patients with neurocysticercosis; however, these trials excluded patients with more than 20 lesions upon brain imaging and had no data in terms of disseminated lesions [5, 6]**.** The evidence regarding the treatment of patients with disseminated cysticercosis, especially those with high lesion load (> 20 lesions), is sparse in the literature. Even the recent guidelines, jointly published by the Infectious Diseases Society of America (IDSA) and the American Society of Tropical Medicine and Hygiene (ASTMH) on the diagnosis and treatment of neurocysticercosis fail to address the issue of diagnosing and treating such patients for lack of evidence [7, 8]**.**

Treatment of disseminated cysticercosis with cysticidal agents was considered hazardous posing a dilemma for the treating physicians/neurologists. In a recent prospective evaluation, besides a comprehensive review of literature, we demonstrated that the standard dose (15 mg/kg/body weight) of albendazole given for 4 weeks was safe in patients with disseminated cysticercosis, and those who received albendazole did better in terms of clinical and radiological outcome as compared to patients who received symptomatic treatment alone. Complete resolution of lesions was observed in 35% of patients, while either reduction in the number of lesions or calcification was observed in the rest of the patients [2]**.** Of late, several authors have initiated and reported good benefit of anti-cysticidal therapy in patients with disseminated cysticercosis [9–14]**.**

We hypothesized that administering 3 cycles of albendazole might lead to the better clearance of neurocysticerci compared to a single cycle. The concept of using more than one cycle was based on the result that a single cycle of 28 days of albendazole in patients with disseminated neurocysticercosis led to complete resolution of only 1/3rd lesions [2]**.** Similar results had been observed in the standard-dose albendazole arm in a previous randomized controlled trial [6]**.** Thus, it seemed appropriate to evaluate the efficacy of 3 cycles of albendazole. It may be noted that a heavy intestinal load of *Taenia solium* eggs usually underlies the phenomenon of dissemination and concurrent taeniasis can range from 16% in patients with mild to moderate infection to more than 80% with heavy infection [15, 16]**.** Classically, the time taken by a juvenile parasite to mature into an adult in the intestinal phase and for the differentiation of an oncosphere to a cysticercus in the tissue phase has been stated to be 3 months and 2–3 months, respectively [16, 17]**.** In order, therefore, to address issues related to reinfection (autoinfection or external reinfection) as well as reactivation of lesions, we spaced the cycles by a difference of 3 months to aid in better clearance of the parasite/lesions.

Our primary outcome measures assessed the efficacy of 3 cycles of albendazole in terms of frequency of seizures and reduction in the quantified intracranial lesion-load in patients with > 20 lesions and those with ≤ 20 lesions. Secondary outcome measures addressed other clinical parameters, laboratory assessment, and clearing of subcutaneous lesions.

## Methods

### Study design and settings

A prospective intervention study was conducted in the Department of Neurology, in collaboration with the Department of Radio-diagnosis, King George’s Medical University, Lucknow, India. This tertiary care University hospital is located in North India and caters to a population of about 100 million. The study period extended from December 2015 to December 2017. Written and informed consent was obtained from every participant; in those with < 18 years of age, parental (or legal guardian) consent was taken for participating in the study. The study was approved by the institutional ethical committee of King George’s Medical University. The trial was retrospectively registered with the ISRCTN Registry bearing the trial registration number ISRCTN11630542.

### Inclusion criteria

All consecutive patients diagnosed with disseminated cysticercosis were included in the study. A diagnosis of cysticercosis was made on the basis of the established diagnostic criteria [18, 19]**.***Disseminated cysticercosis* was defined as the presence of multiple (≥3) cystic viable lesions in the brain, along with evidence of involvement of at least one extra site, like subcutaneous tissues, skeletal muscles, eyes, or presence in any visceral organ [2, 18, 19]**.**

### Exclusion criteria

Patients with disseminated cysticercosis having features suggestive of cysticercal encephalitis were excluded from the study. Cysticercal encephalitis was diagnosed if the patient had signs of raised intracranial pressure, like papilledema, severe headache, altered sensorium, heavy first-contact lesion load, and generalized cerebral edema.

Patients with malignancy, tuberculosis, hepatitis B or hepatitis C virus positivity, human immunodeficiency virus infection, hepatic involvement, focus of any pyogenic infection, and pregnancy were excluded from the study. Patients with a known hypersensitivity to albendazole in childhood, or those who had been administered albendazole, with or without corticosteroids, in the past 6 months were also excluded from the study.

### Patient enrolment and evaluation

Patients attending the neurology out-patient department, with brain imaging suggestive of multiple neurocysticerci, were shortlisted for further evaluation. They were subjected to detailed history and systemic examination, including an examination for subcutaneous nodules/muscle tenderness. Patients presenting with computed tomography of the brain were put through a gadolinium contrast-enhanced MRI of the cranium before moving ahead with other investigations to look for dissemination. Patients with orbital cysticercosis were subdivided based on the involvement of extraocular muscles (myocysticercosis). Patients with vitreoretinal lesions or abutting optic nerve were offered surgical excision prior to initiating albendazole.

**Fig. 1:**
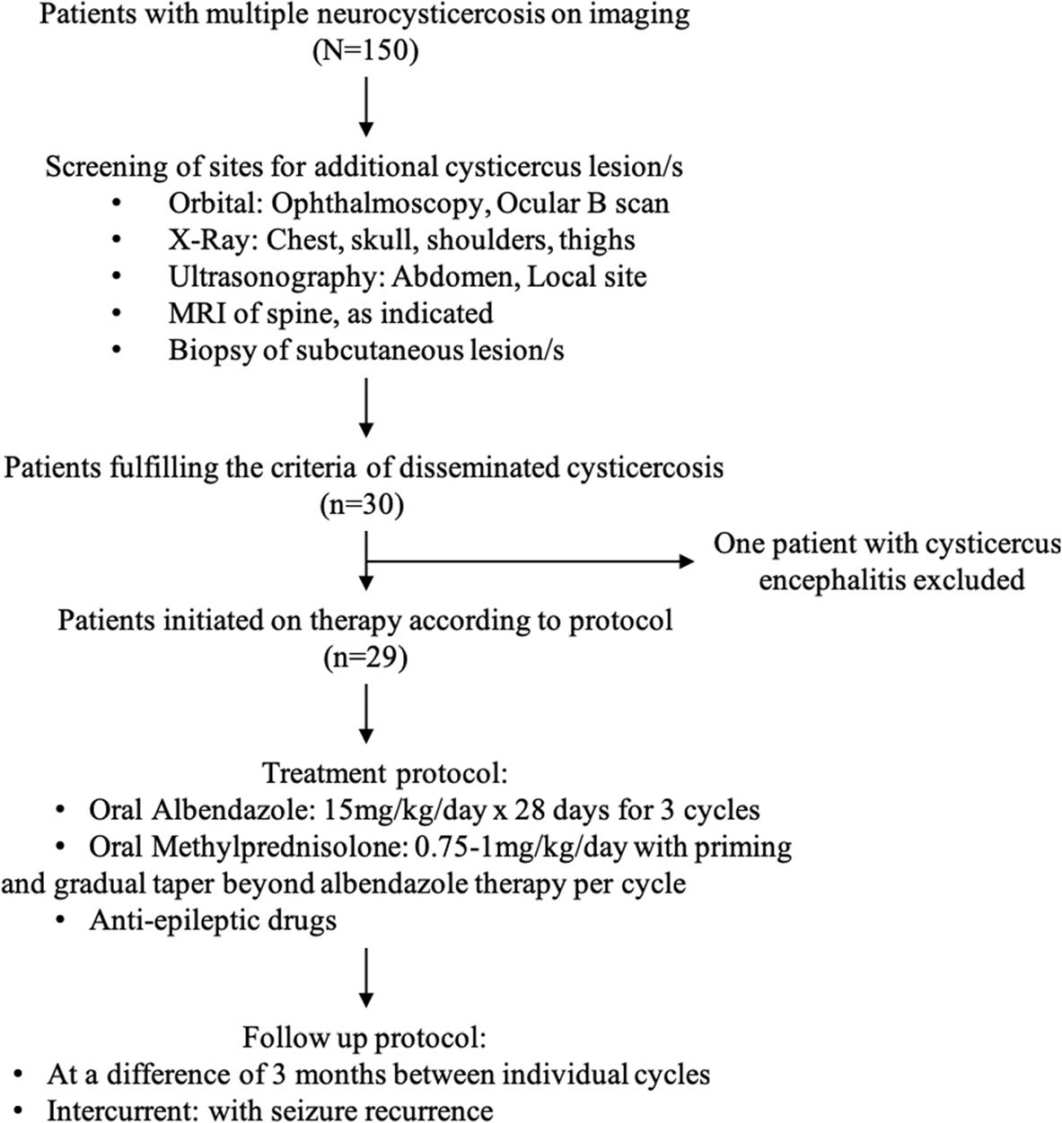
Flow diagram of the study

Subcutaneous nodules detected on detailed survey of the skin were biopsied for histopathological confirmation. Laboratory investigations included a complete blood count, hepatic and renal parameters, glucose estimation, stool examination, and creatine phosphokinase (total) level. Other investigations included direct and indirect ophthalmoscopy, ocular B scan, electrocardiogram/echocardiography, ultrasound of the abdomen, and X-ray of chest, shoulders, hips, and thighs to look for evidence of cysticercal granuloma. Patients who fulfilled the criteria of disseminated cysticercosis were finally included in the study. The flow diagram of the study is shown in Fig. 1**.**

### Neuroimaging

The MRI of the brain was done using a 1.5-T MRI machine (GE Medical system, Sigma Excite Gemsow, USA) with an 8-channel head coil. T1-weighted (T1W), T2W, FLAIR (fluid attenuated inversion recovery), GRE (Gradient recall echo), DWI (diffusion-weighted imaging), and SPGR (spoiled gradient) contrast images were obtained. For contrast study, 0.1 mmol/kg gadolinium (GAD) was injected intravenously. MRI of the spine and whole-body acquisitions were performed when indicated.

In all the 4 different stages observed in patients with neurocysticerci (vesicular, colloidal vesicular, granular nodular, and calcified nodular), the study focused on viable cysticerci and their resolution in response to anti-cysticidal therapy. On the basis of MR imaging, the different stages were defined as follows: *vesicular stage* – lesions with a thin, well-defined cyst wall with contents similar to CSF on T1W and T2W sequences without (minimal, if any) GAD contrast enhancement, scolex defined as a dot iso- or hypo-intense of T1W and iso- to hyperintense on T2W sequences, no perilesional edema; *colloidal vesicular stage* – lesions with a thick and hypointense cyst wall with contents hyperintense on T1W and T2W sequences with GAD contrast ring enhancement, presence of perilesional edema; *granular nodular stage* – lesions similar to those in colloidal vesicular stage but with nodular enhancement and usually with a higher degree of perilesional edema; *calcified stage* – lesions hypointense on T1W, T2W, and FLAIR sequences with blooming on GRE, without (minimal, if any, especially in the early post-seizure phase) perilesional edema, and GAD contrast enhancement. The presence or absence of perilesional edema in different stages was assessed on FLAIR sequence [20, 21]**.**

### Treatment

Patients were administered albendazole after excluding those with cysticercal encephalitis. In patients with vitreoretinal cysticercosis or cysticercal lesions abutting optic nerve, albendazole was initiated at least 6 weeks after the surgical excision of the cyst. Albendazole was given at a dose of 15 mg/kg/day (divided in 2 divided doses). Three days prior to starting albendazole, the patients were primed with oral methylprednisolone (0.75–1 mg/kg of body weight) and it was continued in full dose during the course of albendazole therapy (28 days), followed by tapering in next 2–3 weeks (0.25 mg/kg /week) [2]**.** A total of 3 cycles of albendazole were administered; each cycle was of 28 days and the difference of 3 months between the two evaluations was calculated from the last tapered dose of oral methylprednisolone. The protocol warranted discontinuation of albendazole if any patient developed a rash or an untoward complication like raised intracranial pressure, signs of meningeal irritation, visual disturbances or suggestion of myelitis; oral methylprednisolone, however, was continued to manage the complications arising from the release of antigens. In severe cases, intravenous dexamethasone (0.1 mg/kg of body weight, maximum 30 mg/day), in three to four divided doses for the initial 2 weeks was planned, followed by a gradual taper in the next 2 weeks on stabilization of the patient. Patients in whom stool examination was positive for taenia were not offered any separate treatment, besides the aforementioned albendazole regimen.

Antiepileptic drugs were prescribed to all patients. Oxcarbazepine at a dose of 10-15 mg/kg/day was used as the first-line antiepileptic drug. If the control over seizures was neither obtained nor oxcarbazepine tolerated, levetiracetam was the next drug prescribed. To aid prompt control of seizures, clobazam was added for the initial 2–3 weeks; it was deemed that appropriate therapeutic levels of oxcarbazepine would have been achieved by this time.

### Lesion load assessment

Cysticercal lesion load was assessed at baseline, and subsequently, at each follow-up. Since there are no well-defined methods to quantify the cysticercal lesion load, we devised a radiological assessment protocol to aid comparison of the baseline and follow up parameters, and to avoid an inadvertent under- or over-estimation of load. Lesions were counted at 10 predefined T2-weighted axial levels to appropriately sample the supratentorial as well as the infratentorial compartment of the brain. The levels were defined to ensure a precise count at the given level along with minimization of the false-negative errors attributable to slice-thickness (two levels were not separated by more than one slice-thickness (5 mm) to potentially sample all neurocysticerci). Landmarks in the supratentorial and the infratentorial compartment were basal ganglia-sylvian fissure level and middle cerebellar-peduncular level, respectively. With a single-slice thickness difference, three levels above (supra-ganglionic level, corona radiata and centrum semiovale) and one level below (third ventricular level) the basal ganglia-sylvian fissure level, and two levels above (lower mesencephalon and upper mesencephalon) and two levels below (at the level of inferior olivary nuclei and lower medullary level) the middle cerebellar-peduncular level, were taken into account for objective evaluation of neurocysticerci. All the viable cysts at these levels were counted manually at baseline, and then, at each follow-up. Two authors separately counted the lesions at each level, and any discrepancy was settled by consensus. Coronal and sagittal sections were used for the proper assessment of the axial levels, if there was any discrepancy. Resolution of a lesion at a given site and level in the post-treatment phase was defined by the disappearance of viable cysticerci; degenerating or calcified lesions were not included in the analysis.

### Follow-up and outcome assessment

Patients were assessed thrice after enrolment at a difference of 3 months, each calculated from the completion of an individual cycle. A typical cycle, bordering around 7 weeks, constituted of day 1 to 3 of initial steroid priming, day 4 to 31 of albendazole treatment, followed by an additional 2–3 weeks period of steroid tapering. Outcome assessment was done clinically as well as radiologically. Presence of headache, seizure recurrence and other clinical variables, like focal neurological deficits, vision impairment, and abnormal behavior were assessed in the follow-up period prior to initiation of an individual cycle. Headache was recorded as a dichotomous variable (present/absent), while the occurrence of seizure(s) was recorded both in terms of the proportion of patients as well as the number of seizures sustained. A seizure was noted as partial if consciousness was preserved, or else it was considered as generalized [22]**.** A seizure was recorded as an event if there was a resumption of consciousness in the interictal period; clusters (≥3 seizures in 24 h), similarly, were recorded as an individual event. Occurrence of any adverse drug reaction was also noted in the follow-up period. The radiological outcome was assessed by counting the lesion load at different levels at each of the 3 follow-ups. A subject was labeled as a ‘responder’ if there was > 50% reduction in the lesion load, while ‘failure of treatment’ was defined if even at 3rd follow up the lesion load reduction was < 50%. The response rate was calculated from the baseline cyst-count at each of 1st, 2nd and 3rd visit. Disappearance of all viable cysticerci was termed as ‘complete resolution’. Patients demonstrating either complete resolution or having < 3 cysts at follow up were not offered any further cycle of albendazole.

### Statistical analysis

Statistical analysis was done using the SPSS software version 16.0 (Chicago, IL, USA). The categorical variables were expressed as percentages, while the continuous variables were expressed as median (inter-quartile range) as well as mean ± standard deviation. The Shapiro Wilk test was applied to continuous variables to test the normality of the data. Since most of the continuous variables (lesion load at each level) were not normally distributed, non-parametric tests were applied to compare them. In addition, to provide a gross assessment of change in lesion load, the initial, intervening, and final lesion load were calculated. Initially, the Friedman Test was applied to test the significance of lesion load reduction from baseline to third follow-up at each predefined level. Then, a post-hoc analysis was performed using the Wilcoxon signed-rank test to test the significance of lesion reduction between each follow-up. Seizures occurring through day 1 to 15 of albendazole therapy were discounted. For the over-all comparison, a *P* value < 0.05 was taken as significant, whereas for the post-hoc analysis, a Bonferroni-adjusted significance level was derived in view of multiple comparisons being made. The Mc-Nemar’s test was applied for the comparison of the categorical variables between baseline and follow-up, and the Cochran’s Q test was applied for more than two comparisons.

## Results

### Baseline characteristics

The baseline characteristics of 29 patients with disseminated cysticercosis are shown in Table 1. The mean age of patients was 30.62 ± 15.41 years with a median of 32 years (range: 8–59 years). Twenty-three (79.3%) patients were males. Twenty-five (86.2%) patients were residents of rural area. Fifteen (51.7%) patients were non-vegetarian in their eating habits. The extra-ocular muscles were the most common site of dissemination, involving 8 (27.6%) patients; followed by dissemination to the neck muscles (7, 24.1% patients), vitreoretinal involvement (2, 6.9% patients), and optic nerve (1, 3.4% patient). Out of 8 patients with ocular myocysticercosis, 2 lesions per orbit were present in 2 patients; the rest had one lesion per orbit. Co-morbidities in the form of diabetes mellitus was observed in 2 (6.9%) patients and anemia was present in 1 (3.4%) patient. Seizures (89.7%) were the most common clinical feature, followed by headache (75.9%); abnormal behavior at presentation was seen in 4 (13.8%) patients, vision impairment in 3 (10.3%) patients, and focal neurological deficit in form of hemiparesis in 2 (6.9%) patients. Subcutaneous nodules were detected in 3 (10.3%) patients. Stool examination was positive for taenia in 8 of 29 patients. Four patients underwent a whole-body MRI protocol, while 3 patients were screened to rule out a spinal cord lesion.

**Table 1:** Baseline clinical and demographic profile of 29 patients with disseminated cysticercosis.

S.No	Variables	Value *N* = 29
1.	Age (years)	
	Mean ± Standard deviation	30.62 ± 15.41
	Median	32
2.	Gender	
	Males (%)	23 (79.3%)
	Females (%)	6 (20.7%)
3.	Residence	
	Rural (%)	25 (86.2%)
	Urban (%)	4 (13.8%)
4.	Dietary habits	
	Vegetarian (%)	14 (48.3%)
	Non vegetarian (%)	15 (51.7%)
5.	Site of dissemination	
	Extra-ocular muscles	8 (27.6%)
	Vitero-retinal	2 (6.9%)
	Neck muscles	7 (24.1%)
	Shoulder muscles	1 (3.4%)
	Thigh muscles	4 (13.8%)
	Muscles of mastication	3 (10.3%)
	Optic nerve	1 (3.4%)
	Scalp	1 (3.4%)
	Sub-cutaneous nodules	3 (10.3%)
6.	Comorbid illness	
	Diabetes mellitus	2 (6.9%)
	Anaemia	1 (3.4%)
7.	Clinical features	
	Headache	22 (75.9%)
	Seizure	26 (89.7%)
	• Partial	22 (84.6%)
	• With generalization	4 (15.4%)
	Vision abnormalities	3 (10.3%)
	Abnormal behaviour	4 (13.8%)
	Focal neurological deficit	2 (6.9%)
	Subcutaneous nodule	3 (10.3%)

### Clinical outcome

A significant reduction in the proportion of patients having seizures as well as in the frequency of seizures was observed at individual follow-ups (*P* < 0.001). There was also a significant reduction in the occurrence of headache (*P* < 0.001) (Table 2). A significant difference was not observed in vision impairment, focal neurological deficits, and abnormal behavior. Subcutaneous nodules noted in 3 patients and scalp lesions in 1 patient showed complete resolution.

**Table 2:** Comparison of clinical characteristics at baseline and follow-ups

Clinical features	Baseline (*N* = 29)	1st follow-up (*N* = 29)	2nd follow-up (*N* = 29)	3rd follow-up (*N* = 29)	*P* value
Seizures (Number of patients)	26 (89.7%)	13 (44.8%)	6 (20.7%)	1 (3.4%)	*< 0.001*
Seizures (Number of events)					*< 0.001*
• Total	158	64	18	3	
• Median	4 (2.5–8.0)	0 (0–5.5)	0 (0–0)	0 (0–0)	
• Maximum	15	9	5	3	
Headache (Number of patients)	22 (75.9%)	14 (48.3%)	10 (34.5%)	7 (24.1%)	*< 0.001*

Note: Statistically significant *P* values have been italicized

### Radiological outcome

**Fig. 2:**
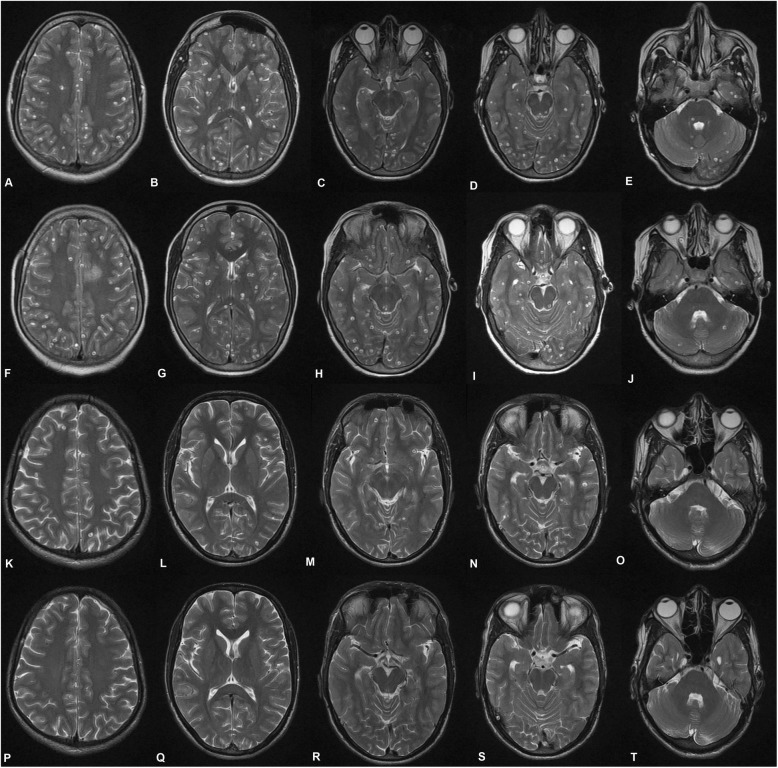
Axial T2W MRI images from a patient of disseminated cysticercosis. **a-e** Baseline images at different levels. **f-j** First follow-up at the same levels. **k-o** Second follow-up at the same levels. **p-t** Third follow-up at the same levels. A graded reduction can be noted from baseline to third follow-up. **a, f, k**, and **p** Centrum semiovale level. **b, g, l**, and **q** Basal ganglia level. **c, h, m,** and **r** First mesencephalic cut. **d, i, n**, and **s** Second mesencephalic cut. **e, j, o,** and **t** Middle cerebellar peduncle level

**Fig. 3:**
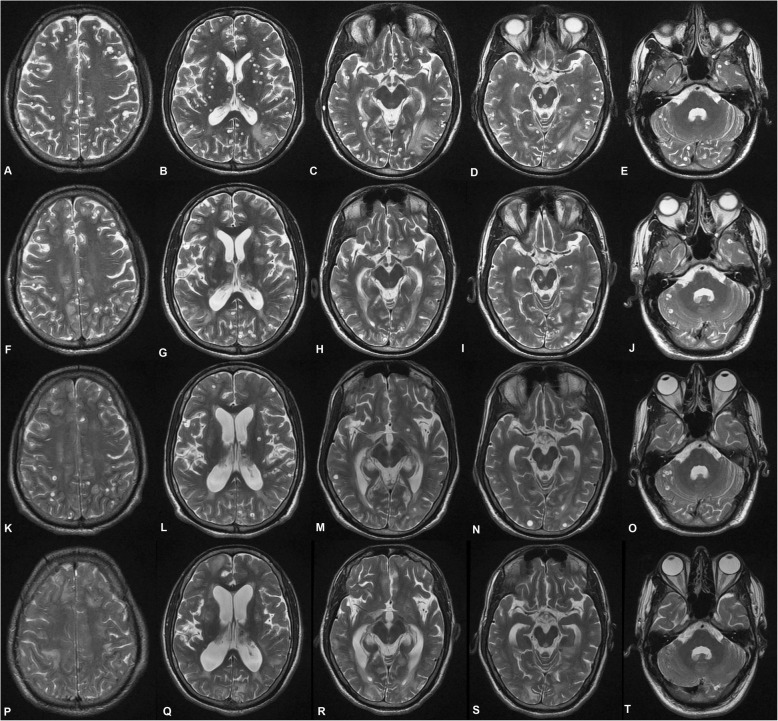
Axial T2W MRI images from another patient of disseminated cysticercosis. **a-e** Baseline images at different levels. **f-j** First follow-up at the same levels. **k-o** Second follow-up at the same levels. **p-t** Third follow-up at the same levels. A graded reduction can be noted from baseline to third follow-up. **a, f, k** and **p** Centrum semiovale level. **b, g, l**, and **q** Basal ganglia level. **c, h, m,** and **r** First mesencephalic cut. **d, i, n**, and **s** Second mesencephalic cut. **e, j, o,** and **t** Middle cerebellar peduncle level

A significant lesion load reduction was noted from baseline to third follow-up at the centrum semiovale level (*P* < 0.001). A post-hoc analysis revealed a significant lesion load reduction between baseline to first follow-up (*P* < 0.001); first to second follow-up (*P* < 0.001); as well as second to third follow-up (*P* < 0.001). A similar reduction of lesion load from baseline to third follow-up was also noted at the first ventricular level, general ventricular level, basal ganglia peri-sylvian level, first temporal cut level, first mesencephalic cut level, and second mesencephalic cut level (*P* < 0.001). The post-hoc analysis revealed a significant reduction of lesion load between baseline to first follow-up (*P* < 0.001); first to second follow-up (*P* < 0.001); as well as second to third follow-up (*P* < 0.001) at all the above-mentioned levels. A collage of the baseline and follow-up images is presented in Fig. 2 and Fig. 3. Bonferroni’s adjustment was applied to the post-hoc *P* values in view of multiple comparisons, but the *P* values remained significant even after correction. A significant reduction in the lesion load from baseline to third follow-up was also seen at the middle cerebellar peduncle, inferior olivary nucleus, and lower medullary cuts (*P* < 0.001). Stratification of lesion load in terms of numbers (≤ 20 or > 20) as well as on the basis of being supra-tentorial or infra-tentorial was also done. Statistically significant reduction was observed within > 20 lesion-load group and ≤ 20 lesion-load group; the median reduction in numbers was 86.3 and 87.5%, respectively (Table 3). Seven of 8 patients in the ≤ 20 lesion-load group did not receive the 3rd cycle of albendazole as their lesion load decreased to < 3. A significant reduction was noted in the lesion load (*P* < 0.001) in the supra-tentorial [baseline: mean = 58.83 ± 99.88, median Inter Quartile Range (IQR) = 25.00 (10.50–43.50); 3rd follow up: mean = 9.31 ± 17.60, median IQR = 3.00 (1.00–8.00)] and the infra-tentorial compartment [baseline: mean = 24.62 ± 38.51, median IQR = 11.00 (4.50–20.00); 3rd follow up: mean = 3.07 ± 7.26, median IQR = 0.00 (0.00–2.00)], with a mean reduction of 84.2 and 87.5%, respectively (in favor of better reduction in infra-tentorial compartment) (Table 4). In terms of response rate, there was no failure in the study. Four patients in > 20 lesions group achieved > 50% reduction in lesion load at the 1st follow up itself. All (≤ 20 and > 20 lesion groups) but one patient (belonging to the ≤ 20 lesion group) achieved > 50% reduction in lesion load from baseline at 2nd follow up. Complete resolution was achieved in 3 patients in the > 20 lesions group while in 6 patients (3 each from ≤ 20 and > 20 lesion groups) the count decreased to < 3 wherefore they were not offered further albendazole treatment.

**Table 3:** Stratified lesion load at baseline and at each follow-up

Number of lesions	Baseline	First follow-up	Second follow-up	Third follow-up	*P* value
**Patients with lesion load >20 (*N*= 21)**					
Total					
Mean ± SD	111.71 ± 153.33	78.1 ± 118.19	47.05 ± 71.62	16.76 ± 27.85	
Median (IQR)	51 (34.5-89)	31 (22.5-68)	18 (8.5-45)	7 (1.5-19.5)	*<0.001*
**Patients with lesion load ≤20 (*N*= 8)**					
Total					
Mean ± SD	9.25 ± 3.85	6 ± 2.62	3.13 ± 1.64	2.63 ± 0.52	
Median (IQR)	8 (7-10.5)	5 (4-8.25)	3 (2-3)^a^	3 (2-3)^a^	*<0.001*

Note: Statistically significant *P* values have been italicized

*IQR* Inter-quartile range, *SD* standard deviation

^a^7 patients with <3 lesions were not given another cycle of albendazole

**Table 4:** Lesions at baseline and 3rd follow-up stratified according to supra-tentorial or infra-tentorial location

Location	Baseline	3rd follow-up	*P* value
Supra-tentorial			
Mean ± SD	58.83 ± 99.88	9.31 ± 17.60	
Median (IQR)	25.00 (10.50–43.50)	3.00 (1.00–8.00)	*< 0.001*
Infra-tentorial			
Mean ± SD	24.62 ± 38.51	3.07 ± 7.26	
Median (IQR)	11.00 (4.50–20.00)	0.00 (0.00–2.00)	*< 0.001*

*IQR* Inter-quartile range, *SD* standard deviation

Note: Statistically significant *P* values have been italicized

### Adverse events

None of the patients experienced any serious adverse drug reaction warranting cessation of treatment. None of the patients experienced the feared complications viz. the development of encephalitis, vision loss, or myelitis after starting treatment. One patient complained of mild itching following the intake of the first dose, his symptoms subsided after giving anti-histamines, and he did not develop similar symptoms with the subsequent doses. Blood counts remained stable during the course of the treatment, while creatine phosphokinase levels showed a mild increase in the levels after the 1^st^ cycle of albendazole. Amongst other parameters, serum bilirubin (predominantly conjugate type) and random blood sugar were found to be elevated during the period of study (Table 5).

**Table 5:** Comparison of biochemical parameters from baseline to third follow-up

Variables (Units)	Baseline *N* = 29	First follow-up *N* = 29	Second follow-up *N* = 29	Third follow-up *N* = 29	*P* value
SGOT (U/L)					
Median (IQR)	41.00 (6.00)	38.00 (5.50)	40.00 (5.50)	41.00 (5.00)	
Mean ± SD	41.00 ± 4.29	39.17 ± 6.50	40.76 ± 5.77	42.14 ± 5.66	0.10
SGPT (U/L)					
Median (IQR)	40.00 (4.00)	40.00 (4.00)	41.00 (6.50)	39.00	
Mean ± SD	39.90 ± 3.58	40.52 ± 5.21	41.28 ± 5.96	(11.00) 41.62 ± 7.82	0.99
ALP (U/L)					
Median (IQR)	78.00 (12.00)	78.00 (6.50)	78.00 (6.50)	72.00	
Mean ± SD	77.69 ± 8.04	79.83 ± 7.79	78.72 ± 6.02	(11.00) 73.62 ± 5.70	0.64
Bilirubin (mg/dL)					
Median (IQR)	1.00 (0.35)	0.90 (0.45)	.90 (0.35)	1.00 (0.40)	*0.016*
Mean ± SD	1.06 ± 0.23	0.89 ± 0.26	0.92 ± 0.32	1.01 ± 0.29	
Urea (mg/dL)					
Median (IQR)	36.00 (7.00)	35.00 (8.50)	40.00 (6.50)	38.00 (5.50)	
Mean ± SD	37.38 ± 3.95	38.48 ± 6.68	39.66 ± 6.61	38.44 ± 5.42	0.82
Creatinine (ng/mL)					
Median (IQR)	0.80 (0.40)	1.00 (0.55)	0.80 (0.40)	0.80 (0.35)	0.98
Mean ± SD	0.86 ± 0.30	0.94 ± 0.35	0.88 ± 0.36	0.91 ± 0.30	
Random blood sugar (mg/dL)					
Median (IQR)	80.00 (11.50)	88.00	89.00 (6.00)	89.00	*0.031*
Mean ± SD	85.76 ± 17.16	(10.00) 92.79 ± 18.40	93.00 ± 15.04	(10.00) 92.28 ± 18.50	

*ALP* Alkaline phosphatase, *IQR* Inter-quartile range, *SD* standard deviation, *SGOT* serum glutamic-oxalacetic transaminase, *SGPT* serum glutamic-pyruvic transaminase

Note: Statistically significant *P* values have been italicized

## Discussion

This was a hospital-based prospective intervention study, evaluating the efficacy of albendazole in patients of disseminated cysticercosis. To the best of our knowledge, quantification of the lesion load reduction, with pre-specified supra- and infra-tentorial levels, following albendazole therapy was done for the first time. A significant improvement in clinical parameters (seizures, headache) and radiologically determined lesion load was seen with 3 cycles of albendazole along with adjuvant corticosteroids and antiepileptic drugs.

Randomized controlled trials have shown that cysticidal agents like albendazole and praziquantel are effective in neurocysticercosis. They were found to be effective in reducing the lesion load and seizure frequency, but these trials have excluded patients with > 20 lesions [5, 6]**.** There have been reports of serious adverse events following cysticidal treatment in disseminated cysticercosis [23]**.** It is worth reiterating that the adverse events are not due to the drug toxicity per se, but rather represent an inflammatory response mounted by the host immune system following massive destruction of cysticerci and the release of cysticercal antigens [23–26]**.**

Two strategies can be used to minimize the risk of untoward effects; the first one requires proper selection of patients while the second one demands the use of corticosteroid adjuvants. The judicious selection of patients is of utmost importance to avoid any adverse event, like blindness. We followed a proper selection strategy before the administration of albendazole; patients were specifically screened for ophthalmological involvement and were given albendazole only after the surgical removal of the cyst. Similarly, the patients with cysticercal encephalitis were excluded as cysticidal agents can cause a massive inflammatory response and even death in these patients [23]**.** Wadia and co-workers reported 3 patients with disseminated cysticercosis who died following the administration of praziquantel. But other authors have reported a favorable response to cysticidal treatment, and no deaths were reported following the treatment [2, 27]**.** We also found a favorable response to albendazole in our patients. We found a significant reduction in seizures as well as headache. We also observed that there was a significant reduction in lesion load after treatment with albendazole. Most importantly we did not observe any major adverse effect, that might have necessitated the cessation of treatment. None of our patients developed complications like encephalitis, blindness, myelitis, or death.

We quantified lesion load by MRI at the baseline and at each follow-up. A reduction in lesion load was observed following each cycle. This type of radiological quantification of lesion load with pre-specified levels in patients of disseminated cysticercosis was done for the first time in this study. Such quantification allows for a correct estimate of lesions, be it supratentorial compartment or the infratentorial compartment, by ear-marking two standard levels in each of the compartment and then covering the brain parenchyma. This strategy automatically corrects errors emanating from studying serial sections by moving either from the medulla below or from the vertex downwards.

Excluding cysticidal drugs and treating the patients of disseminated cysticercosis with anti-epileptics and steroids alone is not fully justifiable. It is likely that few patients may become steroid-dependent and develop re-emergence of symptoms as soon as the steroids are tapered or discontinued [2]**.** It is imperative to state that 3 patients died in the untreated group, with no deaths in the treatment group [2]**.** In our view, such patients with a heavy neurocysticercal lesion load usually land up in a quasi-perpetual cycle of cyst degeneration and antigen release. Such a cycle needs to be broken by cysticidal drugs, otherwise it may not be possible to taper off the corticosteroids anytime in the future course in such patients [2]**.** When the parenchymal lesion load is high, the very unpredictable behavior of cystic degeneration and variable perilesional edema can actually be catastrophic [28]**.**

Corticosteroids are helpful in reducing paradoxical aggravations of inflammation following antiparasitic therapy in patients with neurocysticercosis [29]**.** We administered corticosteroids to all of our patients. Corticosteroids were started 3 days prior to albendazole treatment and were continued at full dose for 28 days, following which they were tapered. Earlier evaluations have used corticosteroids for the duration of the cysticidal therapy with just 1 day of priming or have not elaborated the details of corticosteroid regime [6, 30]**.** It has been shown that the post-cysticidal inflammatory effect, leading to an increase in seizures, is maximally observed during the ensuing 30 days post-treatment [5, 6]**.** In these analyses, the recording of the seizures were censored for 30–60 days post-treatment. We discounted the events only for the first 15 days of albendazole therapy to provide a real-world perspective of anti-cysticidal therapy. We feel that the corticosteroid taper used in our patients basically serves to stabilize the post-cysticidal inflammatory response and it may be said with considerable conviction that the adjuvant use of corticosteroid (priming, concomitant usage, tapering dose) plays a significant role in keeping the complications in check [2]**.** It may be argued that corticosteroid administration, singly, contributed to the clinical benefits observed in terms of seizures and headache. This could have explained the influence only during the initial period of inflammation (early effect) occurring as a result of antigenic release, but not the sustained effect (delayed effect) observed during the follow-up period.

Management of orbital cysticercosis has evolved over the past quarter of a century, and the outcome of such patients has improved considerably. Instead of using the generic term “orbital cysticercosis”, it is advisable to label the patients specifically into those with the involvement of extraocular muscles (orbital myocysticercosis), and with or without the involvement of other structures within the orbit. This dichotomy is extremely useful since most patients with isolated orbital myocysticercosis can be treated successfully with anti-cysticidal therapy, while those having an additional involvement of vitreoretinal space, abutting optic nerve, or proximal to optic canal require a surgical intervention prior to the initiation of anti-cysticidal therapy. To start the oral therapy, we may wait for 6 weeks after the excision, as was done in our patients. Past experiences on the management of orbital cysticercosis have established the role of oral albendazole, with or without praziquantel [31, 32]**.** In these analyses, the patients had uniocular involvement with almost all patients having involvement of a single muscle. In our experience, up to 2 lesions per orbit can be taken safely without the fear of ophthalmic complications. As a corollary, patients with myocysticercosis having ≥ 3 lesions per orbit or > 2 lesions in tandem in a single muscle may not be offered anti-cysticidal therapy.

The exact dose and duration of albendazole therapy in patients with disseminated cysticercosis is not known. An earlier evaluation of patients (*N* = 11) with a heavy load of nonencephalitic cerebral cysticercosis, in fact had 3 patients with systemic involvement i.e. disseminated cysticercosis. Out of 11 patients, 6 patients received either albendazole (for 1 week) or praziquantel (for 1 day), with or without a repeated course; those who received additional anti-cysticidal therapy had better resolution of lesions [30]**.** In our own experience, the resolution of lesions with a single 28-day cycle of therapy had been suboptimal and we, therefore, hypothesized that multiple cycles might be more effective in decreasing the number of lesions further. It may be noted that cyclical albendazole therapy, up to 4 cycles of 4 weeks each with adjuvant corticosteroids, has been shown to be effective in patients with subarachnoid lesions [33]**.** We observed a graded as well as significant reduction in lesion load following each cycle, in both the groups with > 20 lesions and otherwise. We also assessed the difference between the clearance of lesions in the supra-tentorial and the infra-tentorial compartment, and they seemed to clear equally well.

The safety of a longer duration (28 days) of albendazole treatment appears to have been well accepted [2, 33–35]**.** Mild to moderate elevation of enzymes (upto 2–4 times of the upper limit of normalcy) is usually well tolerated by the patients and discontinuation of therapy is warranted either if the levels of enzymes go beyond 4 times or if the patient demonstrates symptoms of hepatic involvement [34, 35]**.** We observed an asymptomatic rise in bilirubin and blood sugar levels in our study. The rise in bilirubin was predominantly conjugated in nature, did not go beyond the range of normalcy, and possibly resulted from a combined effect of albendazole and use of corticosteroids. Blood sugar levels rose but did not qualify for diabetes mellitus. Notably, 2 patients in the study were diabetic at the baseline. In the others, the glycosylated hemoglobin levels were always within the normal limits.

Based on the results of our study, and data thus far available, we may define the group of people with > 20 neurocysticerci where anti-cysticidal therapy might be safe as well as efficacious. The most critical part of including patients with > 20 neurocysticerci is defining the extent of intracranial dissemination, with respect to cysticercal encephalitis, intraorbital involvement and possible obstruction to ventricular flow; and the extent of extracranial involvement, especially with respect to conduction defects or proximity of a lesion to conducting bundles. More often than not, dissemination is *not* suspected, and the patients might be missed. Thus, a good brain MR imaging (preferably done on a 3-T with zero-slice thickness and contrast), ocular ultrasonography, and electrocardiography and echocardiography conjugate, should be done in all patients. Patients with cysticercal encephalitis are best managed with corticosteroids and should not be offered anti-cysticidal therapy. Those with vitreoretinal or orbital apex involvement, and where ventricular obstruction is imminent (along the ventricular foramina) should be offered surgical excision prior to any exposure to anti-cysticidal therapy. We used an interval of 6 weeks between excision and initiation of albendazole therapy in our patients. As a word of caution for patients with extraocular muscle involvement, those with ≥ 3 lesions per orbit or > 2 lesions in tandem in a single muscle may *not* be offered anti-cysticidal therapy; these are difficult situations where even surgical excisions might not be a viable option. Anti-epileptic drugs should be used to control seizures contemporarily as well as those expected with the release of cysticercal antigens, post-therapy. Oxcarbazepine is a good choice in our opinion, however, the use of lacosamide and levetiracetam may be done given the patient profile and issues related to enzyme induction. Priming of patients with corticosteroids before initiating anti-cysticidal therapy is a must and we prefer continuing the corticosteroids for a period of 2–3 weeks (beyond anti-cysticidal therapy) to take care of inflammatory effects caused secondary to release of cysticercal antigens. For patients with < 20 lesions, the clinical practice guidelines given by IDSA and ASTMH should be followed [7]**.**

Being a single-arm open-labeled prospective intervention, it may be contested that a randomized controlled trial might have generated more robust evidence; yes, further evaluation is warranted. In view of paucity of literature on headache characteristics in such patients and the effect of anti-cysticidal therapy on headache frequency and severity, a headache questionnaire may be developed to better quantify the efficacy. To study the long-term effects of anti-cysticidal therapy on the recurrence of seizures a longer (> 2 years) follow-up protocol may be designed.

## Conclusions

Cyclical administration of albendazole appears to be efficacious in patients with disseminated cysticercosis. It is prudent to choose patients judiciously for the albendazole therapy, and corticosteroids must be administered along with albendazole to reduce inflammation resulting from the antigenic release. Radiological quantification at pre-specified levels may help gauge the efficacy of the therapy.

## Data Availability

The datasets generated and/or analysed during the current study are not publicly available due to ongoing work on susceptibility factors and long term outcome, but are available from the corresponding author on reasonable request.

## Abbreviations

ALP: Alkaline phosphatase
ASTMH: American Society of Tropical Medicine and Hygiene
CSF: Cerebrospinal Fluid
DWI: Diffusion-weighted imaging
FLAIR: Fluid attenuated inversion recovery
GAD: Gadolinium
GRE: Gradient recall echo
IDSA: Infectious Diseases Society of America
IQR: Inter Quartile Range
MRI: Magnetic Resonance Imaging
SD: Standard deviation
SGOT: Serum glutamic-oxalacetic transaminase
SGPT: Serum glutamic-pyruvic transaminase
SPGR: Spoiled gradient
SPSS: Statistical Package for the Social Sciences
T1W: T1-weighted
T2W: T2-weighted
